# Insight into the Sporulation Physiology of Elkhorn Fern: Metabolic, Hormonal, and Pigment Changes Within a Single Leaf of *Platycerium bifurcatum*

**DOI:** 10.3390/ijms26168084

**Published:** 2025-08-21

**Authors:** Jakub Oliwa, Iwona Stawoska, Violetta Katarzyna Macioszek, Michał Dziurka, Magdalena Rys, Diana Saja-Garbarz, Anna Maksymowicz, Andrzej Kornaś, Andrzej Skoczowski

**Affiliations:** 1 Institute of Biology and Earth Sciences, University of the National Education Commission, Podchorążych 2, 30-084 Krakow, Poland; iwona.stawoska@uken.krakow.pl (I.S.); andrzej.kornas@uken.krakow.pl (A.K.); andrzej.skoczowski@uken.krakow.pl (A.S.); 2 Laboratory of Plant Physiology, Department of Biology and Plant Ecology, Faculty of Biology, University of Bialystok, 15-245 Bialystok, Poland; v.macioszek@uwb.edu.pl; 3 The Franciszek Górski Institute of Plant Physiology, Polish Academy of Sciences, Niezapominajek 21, 30-239 Krakow, Polandm.rys@ifr-pan.edu.pl (M.R.); a.maksymowicz@ifr-pan.edu.pl (A.M.)

**Keywords:** phytohormones, isothermal calorimetry, Raman mapping, fluorescence microscopy, leaf anatomy

## Abstract

*Platycerium bifurcatum* is one of the most widely cultivated ornamental fern species worldwide and a valuable component of the biodiversity of pantropical forests. In addition to its photosynthetic function, the sporotrophophyll leaves of this species periodically develop a large, clearly demarcated sporangium at the leaf tips, enabling physiological and biochemical measurements both in the active sporulation part and in the non-sporulating leaf area. The aim of this study was to assess anatomical changes, determine thermal effects and the content of selected phytohormones, and analyze the spatial distribution of pigments in the sporophilic and trophophylic part of the same leaf during spore formation. The study utilized fluorescence microscopy, isothermal microcalorimetry, Raman mapping, and ultra-high-performance liquid chromatography coupled with a Triple Quad LC/MS analyzer. The results revealed significant physiological differences between the sporulating and non-sporulating leaf areas. For the first time, differences in thermogenesis within the two leaf regions accompanying sporulation and linked to the sporangium development stage have been demonstrated in ferns. Increases in gibberellins (GA3, GA4, and GA6), auxin (indole-3-butyric acid), (±)-cis, trans-abscisic acid, and abscisic acid glucose ester were observed in the sporophilic part of the leaf, as well as fluctuations in phytohormones in the trophophilic part, indicating internal metabolite relocation within the leaf. Raman analysis and 2D mapping revealed local lignin accumulation and fluctuations in carotenoid levels during spore maturation. The results of this study demonstrate physiological variation within a single leaf and the mechanisms accompanying sporulation, which provide a better understanding of fern adaptive strategies.

## 1. Introduction

*Platycerium bifurcatum* (Cav.) C. Chr. is a species of epiphytic fern that, due to its aesthetic value, is one of the most commonly cultivated ornamental fern species in the world [1,2]. Therefore, a detailed understanding of the mechanisms related to the reproduction of this species is particularly important due to the potential for optimizing production processes. This species originates from Australia and New Guinea [3], where it is a valuable native flora species that is increasingly vulnerable to anthropopressure. At the same time, *P. bifurcatum* can also be a potentially invasive species in urban ecosystems under subtropical climates [4]. This feature is related to its ability to form colonies and its xeromorphic leaf structure, and consequently, relatively high resistance to abiotic stresses [5]. Mature forms produce two morphologically and functionally differentiated types of leaves: sporotrophophylls and nest leaves, differentiated in morphological, anatomical, and functional terms [6]. Sporotrophophyll has a dual function—the trophophilic part participates in photosynthesis, while the sporophilic part is responsible for reproduction through the production of spores. In turn, nest leaves in natural growth conditions (colonies) specialize in specific tasks. The top leaves are then responsible for the uptake of nutrients, while those in the lower sections specialize in water storage. Depending on the place of growth (trees, soil substrate), individuals of *P. bifurcatum* may differ significantly in morphological and physiological terms. In the case of plants grown in the ground, not only an increase in the number of leaves was observed, but also more frequent sporulation than in natural epiphytic forms, which reproduce mainly vegetatively [7].

Sporulation in ferns is a multi-stage process, including cell division, tissue differentiation, the formation of spore-protective structures, and their adaptation to long-term environmental resistance. Thin-spored ferns, which include *P. bifurcatum*, are one of the most morphologically and evolutionarily diverse groups of vascular plants. This diversity is also reflected in generative processes. Insight into the diversity of spore formation mechanisms and the processes that accompany this phenomenon enables a better understanding of adaptive strategies in various ecosystems.

At the cellular level, the process of sporulation involves a complex sequence of mitotic and meiotic divisions and structural changes, the course of which is regulated by both gene expression and hormonal signals [8]. While most studies on hormones focus on seed plants, it is worth emphasizing the key role of phytohormones, including auxins, cytokinins, gibberellins, abscisic acid, and polyamines, in fern development. Gibberellins are involved in initiating spore germination and gametophyte development, especially under light conditions. For example, in *Dryopteris affinis*, gibberellic acid (GA) enhances and supports morphogenetic reactions [9]. Auxins, such as IAA (indole-3-acetic acid), play a key role in cell elongation and pattern formation in emerging gametophytes. Their exogenous administration may, depending on the concentration, support or inhibit the growth of the gametophyte [10].

The initial stage of sporogenesis is the formation of sporangia in the sporophilic parts of sporotrophophyll leaves. Within the sporangium, archesporial cells differentiate, giving rise to sporogenous cells. In ferns, although the genetic sequences differ from those of seed plants, the mechanism of differentiation remains functionally similar [11]. Sporogenous cells divide meiotically, leading to the formation of four haploid spores. A key aspect of sporogenous formation is the synthesis and deposition of a multilayered spore wall rich in biopolymers such as sporopollenin. This substance, partially synthesized in tapetum cells, provides spores with resistance to environmental factors such as UV radiation or dehydration [12]. The final phase of sporulation is spore maturation, including their dehydration and the activation of protective mechanisms. This process depends on hormonal regulation, particularly high levels of abscisic acid (ABA) and other factors that protect against osmotic stress.

Mature spores of *P. bifurcatum* are ellipsoidal and slightly asymmetrically flattened on one side. In the initial phase of development, they are characterized by a green color, which changes to light brown with maturation. The sporoderm consists of two layers: an outer exosporium and an inner endosporium [13]. The surface of the spores exhibits papillary folds and spherical ornamental structures, which may play a role in their dispersal.

In *P. bifurcatum*, sporangia are located in acrostichoids on the sporophilic parts of leaves, located at the dichotomously branched ends of the leaf blade. These structures have a single-layered cell wall and are located on the abaxial side of the leaves, near their axis [14]. The specificity of the arrangement and spatial organization of sporangia makes this species a valuable experimental model in physiological studies. Due to the relatively large, clearly demarcated sporulating area, it is possible to independently measure metabolic parameters both in the zone of active sporulation and in non-sporulating leaf fragments. Such a configuration significantly facilitates the analysis of local physiological processes, which remains difficult in the case of most fern species, in which sporangia are concentrated in numerous, small sores with a small surface area.

Our previous studies on the photosynthetic process during spore formation have shown that the sporotrophophyll leaf is a heterogeneous structure, and the sporulation process in the lower sporophilic part leads to changes in energy absorption and transport in both the upper sporophilic and trophophilic parts of the leaf. Therefore, a deeper insight into the sporulation process of *P. bifurcatum* was conducted. The aim of this work was as follows: (i) assess anatomical changes occurring during the process of spore formation, especially in the sporulating parts; (ii) determine the metabolic activity of the different parts of the leaf during spore formation; (iii) analyze the spatial distribution of chemical compounds, including pigments, in *P. bifurcatum* leaves during sporogenesis; (iv) compare the content of selected phytohormones such as auxins, cytokinins, gibberellins, abscisic acid and jasmonates at individual stages of spore formation and in the trophophilic part of the leaf. For this purpose, in addition to imaging in a fluorescence microscope, isothermal microcalorimetry, Raman mapping, and ultrahigh performance liquid chromatography (UHPLC) coupled with a Triple Quad LC/MS equipped with an electrospray ionization (ESI) source were used.

## 2. Results

### 2.1. Morphology and Anatomy of Sporotrophophylls

**Figure 1:**
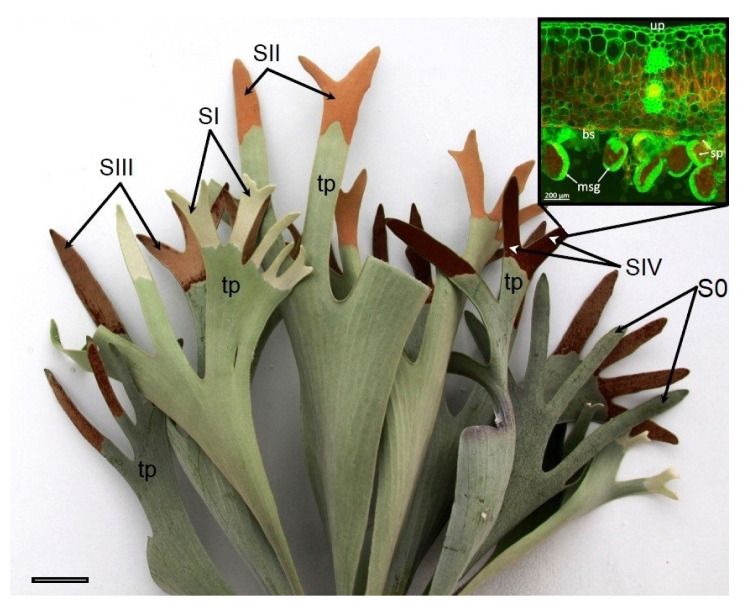
Stages of development of sporangium part on the lower side of *Platycerium bifurcatum* leaf: S0 (stage 0)—non-sporulating leaf, SI (stage I)—stage with young sporangium is characterized by a gray-green color in the area of developing sporangia and the presence of dense layer made of star-shaped trichomes; SII (stage II)—with immature sporangia is characterized by a weakly brown color of leaf tips, the trichomes disappear. SIII (stage III)—leaf tips with immature sporangia are intensely brown. SIV (stage IV) with mature sporangia, is characterized by an intensely brown color of leaf tips and the presence of spilling mature spores; the tips of the leaves are dried. Red color corresponds to the autofluorescence of chlorophyll; tp—trophophilic part of leaf. Abbreviations in the insert: bs—lower side of the leaf, msg—mature sporangium, sp—spores, up—upper side of the leaf. The width of the bar scale = 3 cm.

**Figure 2:**
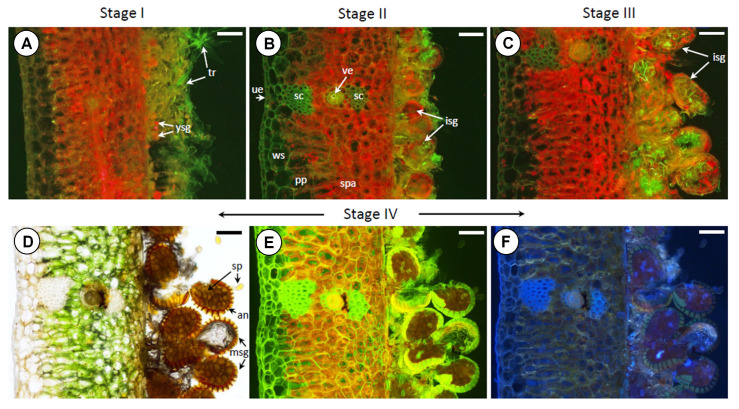
Cross-section of the sporophilic part of the *Platycerium bifurcatum* leaf showing the different stages of development of the sporangium side observed by epifluorescence microscopy. Red and blue colors correspond to autofluorescence of chlorophyll and lignified cell walls, respectively. (**A**) stage I—sporangia are poorly developed, forming a compact layer without visible chlorophyll inside; (**B**) stage II—a visible increase in the size of sporangia, in which chlorophyll appears, stalk cell differentiation, no spores are visible in the sporangia; (**C**) stage III—an increase in the size of sporangia, within which spores can be observed, intense chlorophyll fluorescence is visible in both the stalk cells and the sporangial wall, the sporangial wall is clearly visible, composed of a single layer of cells; (**D**–**F**) stage IV—mature sporangia releasing spores, Chl*a* fluorescence is no longer observed in the sporangial cells, visible increase in lignification of leaf tissues; ue—upper epidermis; ws—water-storage tissue; pp—palisade parenchyma; sp—spores; spa—spongy parenchyma; ve—vein; sc—sclerenchyma; tr—trichomes; ysg—young sporangium, isg—immature sporangium; msg—mature sporangium; an—annulus. The width of the bar scale = 250 μm.

Morphologically different stages of sporangia development in *P. bifurcatum* sporotrophophyll leaves were distinguished based on observations of the lower side of the leaf (Figure 1). Stage 0 is easily distinguished due to the lack of noticeable symptoms of sporangia development. Oliwa et al. [6] provided a detailed description of the anatomical structure of the leaf at this stage. Stage I—the beginning of sporangia development (with a young sporangium) is characterized by a gray-green color in the area of developing sporangia. Stages II and III—with immature sporangia, are characterized by a weakly brown and brown color of leaf tips, respectively. Stage IV—with mature sporangia, is characterized by an intensely brown color of leaf tips and the presence of spilling mature spores. Young leaves are covered on both sides with a very dense layer made of dead star-shaped trichomes, which limits water loss (Figure 1 and Figure 2).

The upper and lower epidermis is made of a single layer of cells with thickened walls, covered with a layer of cutin. Under the cuticle, there is a layer of large, colorless, spherical cells that store water. The deeper parenchyma containing chloroplasts is relatively poorly differentiated anatomically. However, in the dorsal part of the leaf, the cells are palisaded and elongated, while in the ventral part, they are spherical. This tissue is relatively loose, with intercellular spaces. In the central part of the leaf, there is a hadrocentric vascular bundle surrounded by a layer of cells with thickened internal walls. Sporotrophophyll leaves are stiff, thanks to the presence of sclerenchyma located on both sides of the vascular bundle (Figure 2). Young sporangia developing on the lower side of the leaf are hidden in a dense layer of trichomes. A strong chlorophyll *a* (Chl*a*) fluorescence signal indicates an intensive course of photosynthesis in the cells of the developing sporangium (Figure 2A). As they develop further, sporangia set on short stalks become larger and more visible. As they develop, the tomentum becomes increasingly rare (Figure 2B). Also, at this stage, intensive Chl*a* fluorescence is observed. Morphologically, further development of sporangia is associated with an increase in the size of the sporangia, inside which spores can be observed. Intense fluorescence is visible both in the cells of the stalk and the sporangia wall (Figure 2C). At the stage of mature sporangia set on stalks, the sporangium wall is clearly visible, built of one layer of cells with strongly thickened walls forming a ring (annulus), and mature, brown spores are visible inside. Mature sporangia burst, releasing spores. At this stage of sporangia development, Chl*a* fluorescence is no longer observed in the cells building the sporangium (Figure 2D–F).

### 2.2. Isothermal Calorimetry Analysis

**Figure 3:**
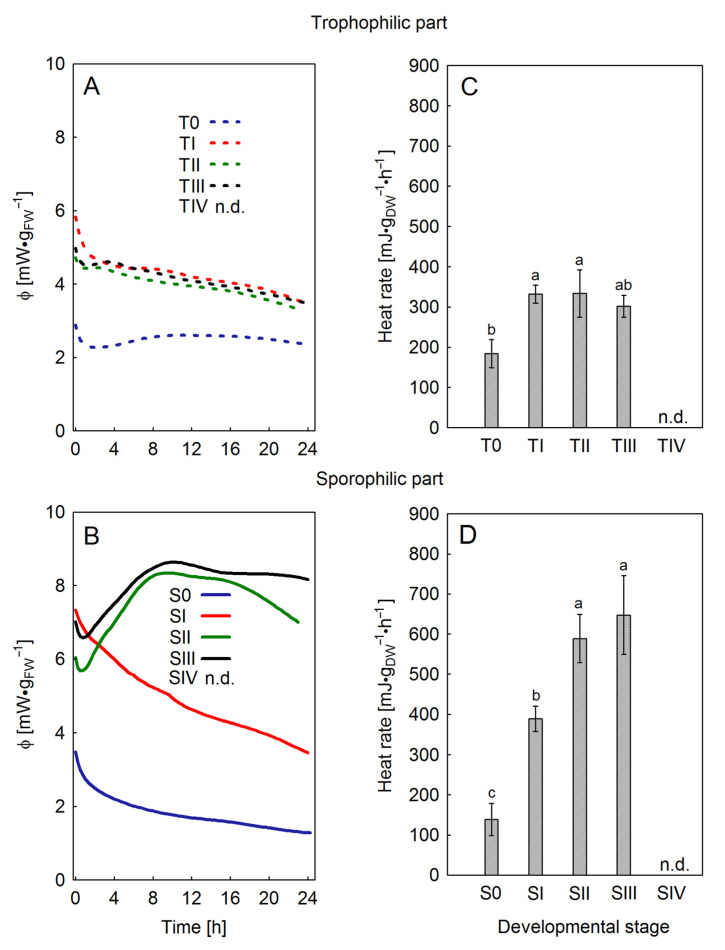
Specific heat flow (**A**,**B**) and heat rate (**C**,**D**) in the sporophilic and trophophilic parts of *Platycerium bifurcatum* leaves before (stage S0, T0, respectively) and during the sporulation (stage SI‒SIV, TI‒TIV, respectively); n.d.—not determined. Mean values from 5 biological replicates ±SD, marked with different letters, differ significantly according to Duncan’s test, *p* ≤ 0.05.

Significant differences in the emission of thermal power and thermal energy during the sporulation were demonstrated between the sporophilic and trophophilic parts of the leaf. It should be emphasized that the values of the mentioned parameters are directly proportional to the metabolic activity of the tissue [15]. Both the shape of the thermal power curve and heat rate in the trophophilic part did not differ significantly in the TI–TIII stages (Figure 3A,C), but they had higher values than in the nonsporulating leaves (T0). In the sporophilic part (Figure 3B,D), anatomical and physiological changes related to the formation of sporangia caused a significant increase in the heat rate between S0 and SI, SII, and SIII. A change in the shape of specific thermal power curves was also observed between the SI and SII stages, with a significant increase in emission occurring approximately 8 h after the start of measurement. Stage IV (SIV) was almost entirely composed of dead tissue; therefore, calorimetric measurements were pointless.

### 2.3. Phytohormone Profile

**Figure 4:**
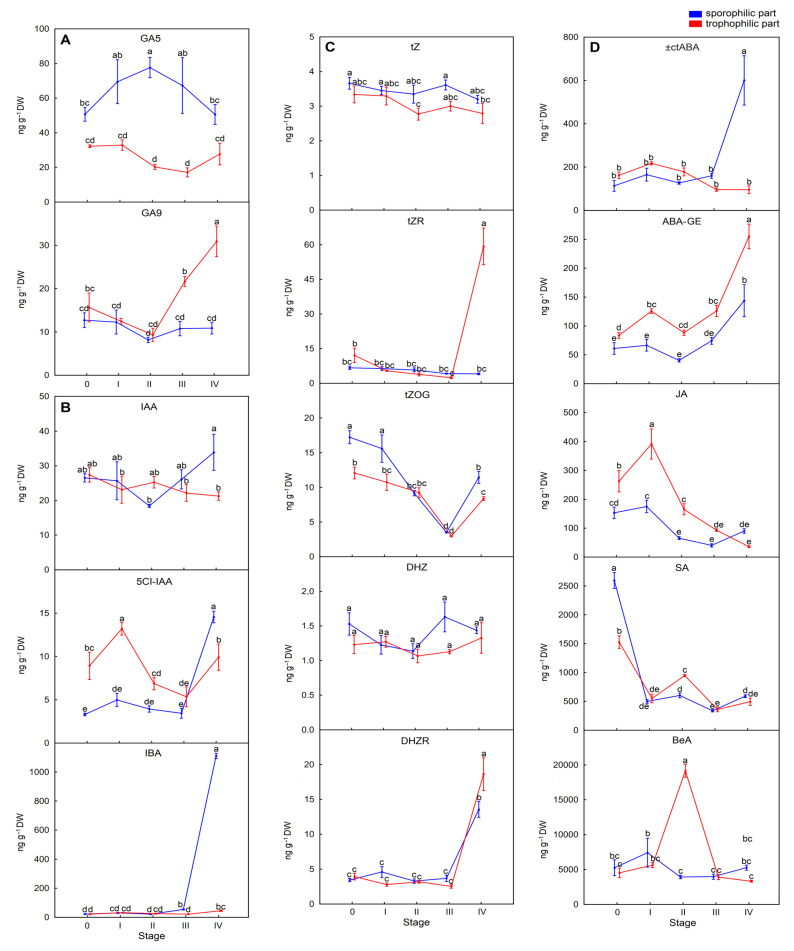
Content of selected phytohormones in the sporophilic and trophophilic parts of *Platycerium bifurcatum* leaves before (stage 0) and during the sporulation (stage I–IV). (**A**) Gibberellins: GA3, GA5; (**B**) auxins: indole-3-acetic acid—IAA), 5-chloroindole-3-acetic acid (5-ClIAA), indole-3-butyric acid (IBA); (**C**) cytokinins: trans-zeatin (tZ), trans-zeatin riboside (tZR), glucosylated trans-zeatin (tZOG), dihydrozeatin (DHZ), dihydrozeatin riboside (DHZR), (**D**) (±)-cis, trans-abscisic acid (ABA) abscisic acid glucosyl ester (ABA-GE), jasmonic acid (JA), salicylic acid (SA), benzoic acid (BeA). Mean values from 3 independent biological replicates presented in a single graph and marked with different letters differ significantly according to Duncan’s test, *p* ≤ 0.05.

A few gibberellins (GAs), sporulation-related phytohormones, were detected in both parts of sporotrophophylls with different content patterns at each sporulation stage (Table S1). In the trophophilic part, the total GA content was significantly lower than in the sporophilic part. The total GAs increased by 79.8% at SIV in comparison with S0 as sporulation progressed in the sporophilic part, and their content correlated with the maturation of sporangia and spores (Table S1). The most abundant in the sporophilic part was active gibberellin GA3 and precursor one GA5. In the trophophilic part, GA3 was detected only at SIV (Figure 4). As an exception, GA9 content was higher in the trophophilic part at SIII and SIV than in the sporophilic part (Figure 4A), indicating that this gibberellin affected the physiological activities in the trophophilic part of a leaf.

The content of one of the most potent plant hormones, an auxin indole-3-acetic acid (IAA), was similar in both parts of sporotrophophylls at all investigated sporulation stages (Table S1). However, two other detected auxins, 5-chloroindole-3-acetic acid (5-ClIAA) and indole-3-butyric acid (IBA), exhibited different content patterns in the trophophilic and sporophilic parts of a leaf, depending on the sporulation stage (Figure 4B). The content of 5ClIAA was significantly higher in the trophophilic part than in the sporophilic part at the S0‒SIII stages, but it gradually decreased in both parts of the leaf from SI. Then, 5-ClIAA content increased in both parts of a leaf at SIV, showing a significantly higher content in the sporophilic part (Figure 4). In the case of IBA, its content oscillated at similar levels in both parts of a leaf at S0-SIII. Still, it suddenly increased significantly over 20 times (from 22.68 ng g^−1^ DW at S0 to 1111.16 ng g^−1^ DW) only in the sporophilic part at SIV when the sporangia matured and spores were released (Figure 4B). At this stage, the total auxin content also strongly increased by 31.3% in the trophophilic part compared with S0 (Table S1, Figure 4B).

The active cytokinins, including trans-zeatin (tZ), cis-zeatin (cZ), dihydrozeatin (DHZ), and N6-isopentenyl adenine (iP), were detected in both parts of sporotrophophylls at all sporulation stages (Table S1). Both tZ and DHZ contents oscillated at comparable levels in both investigated parts of a leaf at all stages, with one exception. Only in the sporophilic part at SIII, both tZ and DHZ contents were significantly higher compared to the trophophilic part but not to the controls (Figure 4C). However, cZ, iP, and their riboside contents did not differ between the two parts of the leaves, as well as between developmental stages (Table S1). Interestingly, glucosylated trans-zeatin tZOG content was significantly higher in the sporophilic part than in the trophophilic part at all developmental stages, but SIII (Table S1).

In the case of the ribosides tZR and DHZR, their content also oscillated at similar levels in both parts of sporotrophophylls. Surprisingly, a significant increase (approximately 5 times) in the tZR content was observed only in the trophophilic part at the final developmental stage (SIV) (Figure 4C). Furthermore, the content of DHZR significantly increased about 3 times in the sporophilic and 5 times in the trophophilic part at the SIV stage compared to the earlier developmental stages. Additionally, aromatic cytokinin meta-topolin riboside (mTR) was detected in the trophophilic part only at SI and in the sporophilic part only at SII (Table S1).

The average contents of the total auxin and the total cytokinin indicated that auxin content (primarily because of IBA content) was significantly higher in the sporophilic part of a leaf at SIV, with a 14.9 ratio of auxins to CKs. Moreover, the total CK content showed a decreasing trend, significantly below the values observed at S0 in the sporophilic part. In the case of the trophophilic part, a similar tendency was noted as in the sporophilic part, except for an increase of total CKs by 36.6% at SIV (Table S1).

Besides these three phytohormone groups described above, several acidic phytohormones, such as (±)-cis, trans-abscisic acid (ABA) and its glucosyl ester (ABA-GE), jasmonic acid (JA), salicylic acid (SA), and benzoic acid (BeA), were detected (Figure 4D). However, these phytohormone contents were at a similar level or higher in the trophophilic part at SI-SIII than in the sporophilic parts. Only at SIV, the contents of ABA, JA, and BeA were significantly (*p* < 0.05) higher in the sporophilic part compared to the trophophilic part, indicating that they play a role at the final stage of the proper spore maturation. Moreover, both forms of abscisic acid increased gradually in sporophilic parts with sporulation progression compared to the control (Figure 4D). The content of JA and SA showed a decreasing tendency in both parts of sporotrophophylls (Figure 4D). In the case of benzoic acid, which is a common compound in ferns, its content was high in both the trophophilic and sporophilic parts at the investigated developmental stages. A significant increase (approximately 5 times) in its content was observed only in the trophophilic part at SII. At stages SIII and SIV, BeA decreased to a level below that of the control content in both parts of a leaf (Figure 4D).

### 2.4. FT-Raman Analysis

**Figure 5:**
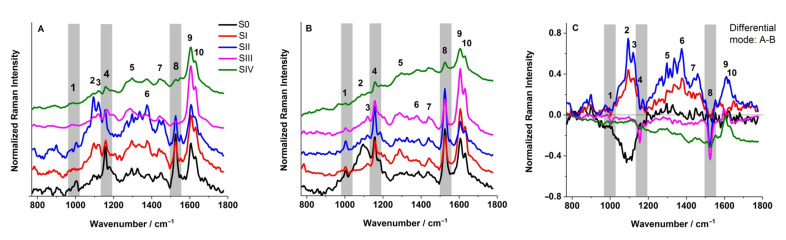
Normalized FT-Raman spectra of the bottom (**A**) and upper (**B**) sides of sporophilic parts of *Platycerium bifurcatum* leaves. (**C**) Presents a differential mode of (**A**,**B**) spectra. The numbers assigned to the particular peaks are explained in the text, Table S2. Spectra represent mean values from 6 to 10 replications.

The identification of chemical compounds assigned to the particular bands in the FT-Raman spectra obtained from the bottom and upper sides of the sporophilic part of *P. bifurcatum* leaves is presented in Table S2. Furthermore, the differences in the following sporulation stages of the tested leaves relating to the main components are clearly illustrated in Figure 5. The most characteristic bands are derived from carotenoids (Car) at 1005, 1159, and 1525 cm^−1^. The peaks observed at 1159 and 1525 cm^−1^ are attributed to -C-C- (peak no. 4) and to -C=C- (peak no. 8) stretching vibrations of the polyene chain, respectively. Furthermore, the position of band no. 8 suggests that the Car moiety possesses nine conjugated -C=C- bonds [16]. The third, low-intensity band at 1005 cm^−1^ (Figure 5—peak no.1) reflected -CH_3_ groups attached to the main chain coupled with -C-C- bonds [17,18,19]. The peaks at 1093 and 1121 cm^−1^ arise from the polysaccharides (the asymmetric C-O-C, 1093 cm^−1^, and the symmetric C-O-C, 1121 cm^−1^ vibration), whereas the presence of lipids and fatty acids is detected at 1293, 1375, 1440, and 1631 cm^−1^ [18]. All the examined plant tissues have shown bands specific to chlorophylls, as well as to polyphenols (1606 cm^−1^) [18,19]. The intensity of this band is undoubtedly influenced by the presence of lignin found in the walls of developing sporangia.

Lignin is a polymer composed of derivatives of phenolic alcohols, including coniferyl alcohol, sinapyl alcohol, and p-coumaryl alcohol. Notably, during successive stages of leaf development, the relative Raman band intensities at approximately 1606 cm^−1^ and 1525 cm^−1^—characteristic of polyphenolic compounds and Car, respectively—exhibited significant changes. The observed increase in the intensity of the 1606 cm^−1^ band, accompanied by a simultaneous decrease in the intensity of vibrations associated with the –C=C– stretching mode of the polyene chain, strongly indicates the progressive development of sporangia, a concurrent decline in Car pigment content, and a probable increase in lignin accumulation within the walls of developing sporangia. These spectral changes are most pronounced on the abaxial (bottom) leaf surface—corresponding with the localization of sporangial formation—where the I(1606)/I(1525) ratio increases markedly from 0.85 at stage T0 to 2.09 at the stage SIV (Figure 5A). In contrast, the signal obtained from the adaxial (upper) leaf surface exhibits considerably less variation, with the I(1606)/I(1525) ratio changing from 0.86 to 1.25 across the same developmental stages (Figure 5B).

The recorded signal for stages from T0 to SIII on the adaxial (upper) leaf surface is markedly more intense, particularly within the spectral range associated with the carotenoid triplet (Figure 5B). This enhancement reflects the anatomical characteristics of the leaf. In contrast, the presence of sporangia on the abaxial (bottom) surface may hinder signal acquisition from the underlying leaf tissue. Moreover, chemical constituents associated with sporangia development—most notably lignin, as reflected by band no. 9—are more prominently represented in spectra acquired from the bottom leaf side (Figure 5A).

To clearly illustrate the spectral differences between the lower (Figure 5A) and upper (Figure 5B) surfaces of *P. bifurcatum* leaves, a differential spectrum was generated (Figure 5C). This analysis highlights significant differences in signal intensity, particularly at frequencies corresponding to variable concentrations of sugar-related compounds (peaks 2 and 3), carotenoid pigments (peak 8), phenolic substances (peak 9), as well as lipids and fatty acids (bands 5, 6, and 7). These distinctions are especially pronounced in stages SI and SII.

**Figure 6:**
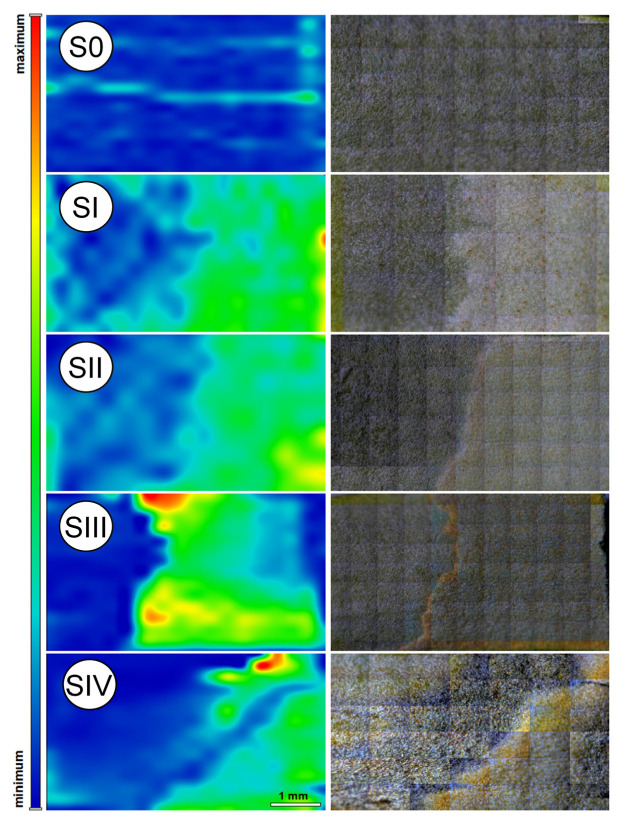
2-D Raman maps of *Platycerium bifurcatum* leaf (sporophilic part) in their various sporulation phases: S0—non-sporulating leaf; stage I (SI) leaf with young sporangium; stage II and III (SII and SIII) respectively, leaves with immature sporangium; stage IV (SIV)—leaf with mature sporangium. The grey panel (the right panel)—raw photos from the apparatus. Colored panel (the left one) presents integrated bands related to -C=C- stretching vibration modes (1525 cm^−1^) and painted according to carotenoids content (blue—minimum content, red—maximum content). At stage S0, the Car concentration was relatively low and homogeneously distributed across the mapped area. In subsequent stages (SI and SII), pigment levels increase and remain comparable between these stages, with enhanced localization in the sporophilic parts, green areas in Figure 6. Transition to stage SIII—corresponding to spore maturation—was marked by an insignificant reduction in the overall surface area expressing carotenoid signals, alongside the emergence of distinct high-intensity zones (represented in yellow and red). These hotspots are likely associated with intersporangial tissues undergoing localized pigment accumulation. The border between trophophilic and sporophilic tissue becomes significantly more defined. Finally, the Raman map of the leaf in stage SIV reveals almost a complete loss of carotenoid-associated signal, reflecting the presence of fully matured spores and the onset of desiccation in living tissues.

Raman 2D chemical mapping of the abaxial surface (Figure 6) further delineates the boundary between the trophophilic and sporophilic parts of the leaf. When analyzing the mapped chemical profiles across sporulation stages from S0 to SIV, progressive changes in Car distribution become evident. As previously mentioned, spectral bands associated with –C=C– stretching vibrations (at 1525 cm^−1^) were used to visualize Car content, and they are presented as color-coded 2D maps (Figure 6).

### 2.5. Chlorophyll and Flavonoids Content

**Figure 7:**
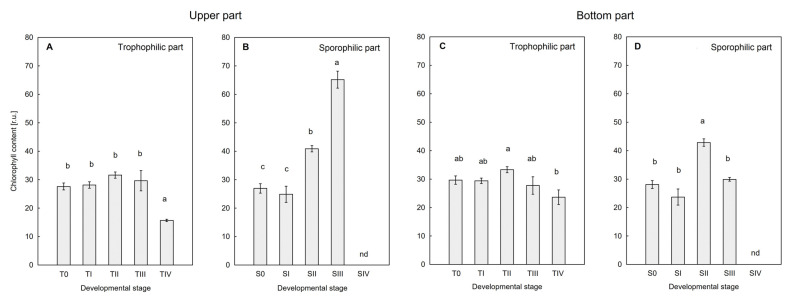
Changes in chlorophyll content in trophophilic (**A**,**C**) and sporophilic parts (**B**,**D**) of *Platycerium bifurcatum* leaf were measured non-destructively on the upper and lower sides of the leaf blade, in subsequent sporulation stages SI‒SIV/TI‒TIV and in a non-sporulating leaf (S0/T0); nd—not determined. Mean values from 6 independent biological replicates, different letters indicate significant differences according to Duncan’s test, *p* ≤ 0.05.

Based on the analysis of the chlorophyll in the leaves, it was shown that the Chl content at the SIV stage significantly decreased in the upper trophophilic part of the leaf blade (Figure 7A) in comparison with the others, in which the level was comparable. In the sporophilic part (Figure 7B), the Chl content at the S0 and SI stages was identical, and a gradual increase of this dye was initially observed at stage SII and then also at SIII, where its value was about 65 au. Due to the large amount of lignification of the sporophilic part of the leaf at the SIV stage, the Chl content was unmeasurable (Figure 2). In the trophophilic part on the bottom side of the leaf blade (Figure 7C), a statistically significant difference in the Chl content was only found between stages SII and SIV. In the sporophilic part, on the other hand (Figure 7D), a significant increase in the Chl content was observed, similar to the trophophilic part at the SII stage. However, this regularity was not sustained at stage SIII. The Chl content in the sporophilic part on the bottom side of the SIV leaf blade was not measurable.

**Figure 8:**
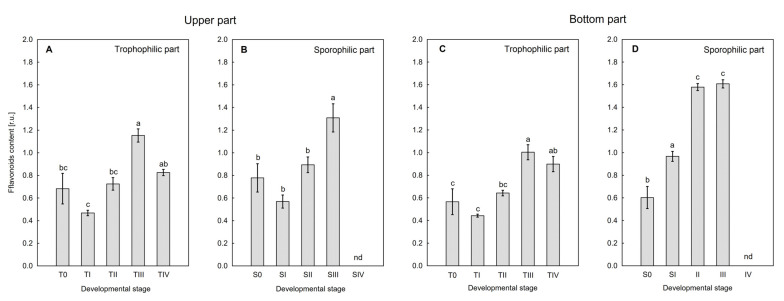
Changes in flavonoid content in trophophilic (**A**,**C**) and sporophilic parts (**B**,**D**) of *Platycerium bifurcatum* leaf were measured non-destructively on the upper and lower sides of the leaf blade, in subsequent sporulation stages SI‒SIV/TI‒TIV and in non-sporulating leaf (S0/T0); nd—not determined. Mean values from 6 independent biological replicates, different letters indicate significant differences according to Duncan’s test, *p* ≤ 0.05.

It was shown that the content of flavonoids (Flav) in the trohophylic part of both the upper (Figure 8A) and bottom side (Figure 8C) of the leaves varied similarly. A significant increase in the Flav content was observed at stage SIII relative to earlier stages, S0, SI, and SII. In the sporophilic part on the upper side of the leaf blade, the Flav content also increased rapidly at stage SIII (Figure 8B). However, on the bottom side, this increase was already observed at stage SII, and remained at a similar level also at stage SIII (Figure 8D). Due to the large lignification of the sporophilic part of the leaves in the SIV stage, the content of Flav on both the upper and bottom sides of the leaf blade was not measurable.

## 3. Discussion

Four developmental stages can be distinguished in the sporulation process of *P. bifurcatum* [20]. This publication presents the morphological, anatomical, and physiological changes that occur from stage 0 (no sporulation features) to the release of spores in stage IV. The anatomical presence of a hadrocentric vascular bundle and extensive layers of sclerenchyma in the sporotrophophylls of *P. bifurcatum* indicates conservative strategies of mechanical and vascular enhancement in different fern species [21]. A typical feature of *P. bifurcatum* is the presence of strongly developed trichomes at SI in the sporophilic parts located at the terminals of the sporotrophophylls, which protect the spores at the early stages of development. Such adaptations, although also found in other epiphytic ferns, are much rarer in terrestrial species [22]. The dense covering of trichomes may play a role in the process of sporogenesis, protecting the tissue from water loss and excessive solar radiation and promoting microenvironmental stability around developing spores. These structures also have the effect of reducing transpiration and mechanically shielding the developing spores.

As we demonstrated earlier [20], the onset of sporulation of *P. bifurcatum* was associated with increased photosynthetic activity, especially in the sporophilic part of the leaves. The following stages of sporulation were accompanied by pigmentation changes in both the sporophilic and, to a lesser extent, the trophophilic parts of the leaf. Also, in other species such as *Adiantum pedatum* or *Pteridium aquilinum*, changes in the leaf pigment and thickening of the sporangial walls have been noted during sporulation [23]. Fluctuations in chlorophyll content were related to the photosynthetic activity of the leaf during sporogenesis. In the sporophilic part, an increase in Chl content was observed at the SII and SIII stages on the upper side of the leaf. At the same time, analysis of heat rate and power curves confirmed the increase in metabolic activity of the tissue. In the present study, the isothermal microcalorimetry method was employed for the first time to illustrate metabolic processes occurring during spore formation in ferns. The similarity of the curves and values of specific heat power in trophophilic and sporophilic parts of leaves at S0–SI stages suggests physiological homogeneity before the onset of sporogenesis. A significant increase in the value of specific heat power was recorded only in the sporophilic parts at stages SIII–SIV, which emphasizes the connection of increased metabolic activity with spore formation and maturation. Local thermogenesis may reflect increased energy requirements for processes such as sporoderm formation, lignification, or acquisition of dehydration resistance [14].

In many fern species, the development of spores is localized in sori scattered on the underside of assimilatory leaves, which makes it challenging to study the sporulating and non-sporulating parts separately [14,24]. In contrast, in *P. bifurcatum*, the spores are located in a large, compact area at the terminals of the sporotrophophylls, which allows them to be clearly distinguished by developmental stage and spatially separated from the rest of the leaf. These features allow for a more precise analysis of not only metabolic but also structural changes that occur during the formation, maturation, and release of spores.

Anatomical cross-sections of selected leaf fragments (Figure 2), combined with fluorescence imaging of the investigated plant tissues, provide strong corroborative evidence for the changes observed in the FT-Raman spectra (Figure 6) and the Raman mapping results (Figure 7). A comparative analysis of the fluorescence images (Figure 2), which correspond to tissues sampled in stages SI, SII, SIII, and SIV, respectively, clearly confirmed dynamic alterations in the content, spatial distribution, and composition of pigments within the analyzed leaf tissues. A progressive shift in pigment content is apparent when comparing sections from stages SI to SIII (Figure 2A–C). In stage SI, the emergence of a developing trichome layer is evident, while stage SII reveals the formation and early development of sporangia. Figure 2C, corresponding to stage SIII, reflects compositional changes in the pigment profile, accompanied by an increase in Car accumulation. In contrast, stage SIV (Figure 2D–F) exhibits only minimal fluorescence associated with Chl and Car, which can be attributed to tissue desiccation during the final stages of spore formation.

All these morphological and anatomical features, as well as changes in pigment composition and the process of photosynthesis in *P. bifurcatum*, are under the control of endogenous phytohormones, whose levels differ in the sporophilic and trophophilic parts of a leaf during sporulation. The most influential phytohormones during sporulation and spore germination in ferns are gibberellins [25,26]. In this manuscript, phytohormone profiling of sporotrophophylls confirmed that sporulation depends primarily on a gradual and progressive increase in total gibberellin content, resulting in an almost 80% increase at the SIV stage in the sporophilic part of a leaf. However, among the five gibberellins detected in the sporophilic part, only biologically active GAs GA3, GA4, and GA6 showed enhanced levels at the final mature spore stage (Figure 4A, Table S1). In sporophytes of the other epiphytic fern *Asplenium nidus*, gibberellins GA3, GA4, and GA9 were also detected, with a particularly high level of GA4 [27]. Moreover, the processes of spore maturation and release appear to rely also on increased levels of the auxin IBA, as well as ±ctABA and ABAGlc in *P. bifurcatum* (Figure 4B,D). Elevated levels of GA4 and ABA are consistent with previous research on phytohormone profiles in sporophytes of tree ferns such as *Cibotium glaucum* and *Dicksonia antarctica* [28].

In contrast, in the trophophilic part of a leaf, responsible for extensive photosynthesis and nutrient supply for the sporophilic part, GA3 and GA6 were not detected in the case of most sporulation stages. However, GA_3_ and increased GA9 levels were observed at the spore release stage (SIV) in this part of the leaf (Table S1). Additionally, IBA and only ABAGlc levels significantly increased in the trophophilic part; however, their contents were not as elevated as in the sporophilic part (Figure 4B,D). We speculated that ABAGlc, as it is a transported form of ABA, was transferred from the trophophilic part to the sporophilic part and hydrolyzed there to achieve a high level of ABA, supporting the process of spore dehydration and protecting them against osmotic stress [29].

It has to be emphasized that although various types and forms of cytokinins were detected in both parts of sporotrophophylls, including tZ, cZ, DHZ, IP, and their ribosides, the total CK content significantly decreased below the control levels at all sporulation stages (Figure 4C, Table S1). This phenomenon suggests that cytokinins are not extensively involved in sporulation, particularly in spore maturation and release in *P. bifurcatum*. The phytohormone profile of the fern-like vascular plant *Psilotum nudum* (belonging to *Pteridophyta*) revealed that from all detected cytokinins, only DHZR was increased at the final sporulation stage [30], similar to sporotrophohylls of *P. bifurcatum*. Moreover, an application of exogenous cytokinins retarded spore germination in *Dryopteris filix-mas* [31]. Reduced cytokinin contents, together with the elevated level of ABA, can be related to the initiation and progression of senescence and dying of the trophophilic part, which at the SIV stage is almost entirely dead, and to a lesser extent also of the sporophilic part of a leaf. Furthermore, ABA-dependent regulation may also be responsible for chlorophyll breakdown during the final stage of sporulation [32]. All these catabolic processes were supported by a gradual, significant reduction of JA and SA levels observed in both parts of sporotrophophylls (Figure 4D, Table S1).

Raman spectroscopy offers a rapid, non-destructive, label-free, and often in-field applicable method for analyzing the biochemical composition of plants. Traditional analytical methods often require destructive sampling, solvent extraction, or chemical labeling, limiting temporal resolution and spatial precision. Raman spectroscopy bypasses these constraints, offering non-invasive molecular profiling of intact plant tissues. By capturing molecular fingerprints through vibrational spectra, it enables the real-time monitoring of physiological responses to environmental stressors, pigment composition, and protein profiles [33,34,35].

The FT-Raman spectra (Figure 5), Raman imaging maps (Figure 6), and fluorescence microscopy images (Figure 2) collectively present a coherent dataset. The results obtained using these complementary techniques demonstrate internal consistency in most sporulation stages in *P. bifurcatum* leaves. However, some discrepancies were observed between the FT-Raman spectra (collected from the abaxial leaf surface) and the corresponding 2D Raman images, particularly for developmental stage S0. Figure 5A indicates a relatively high Car content not only in stages SI and SII, but also in the initial stage S0, as evidenced by the presence of characteristic peaks (bands 1, 4, and 8). In contrast, the 2D Raman image of the same stage (Figure 6) reveals a uniformly low Car distribution across the mapped area, which is entirely represented in blue.

This discrepancy can be reasonably attributed to the fundamental differences in data acquisition methods. While the 2D Raman maps represent spatially resolved surface imaging, the FT-Raman spectra were acquired from discrete, localized points subjected to focused laser irradiation. It is therefore plausible that, in the stage S0, the point chosen for spectral acquisition coincided with a region exhibiting anomalously low Car concentrations. Another potential explanation involves sampling errors related to tissue type: in stage S0, the spectrum may have been inadvertently recorded from a trophophilic part, which typically contains higher levels of photosynthetic pigments, rather than the sporophyllous tissue. This is particularly likely given the absence of distinct morphological features that differentiate these tissue types at early sporulation stages. Despite these minor methodological discrepancies, all employed techniques consistently confirm that the content of photosynthetic pigments in the analyzed tissues is closely linked to the sporulation stage. This, in turn, reflects the progression of sporangium formation and spore maturation.

The decrease in Chl content in the trophophilic part (upper blade) in SIV is associated with a decrease in photosynthetic activity and chloroplast degradation, senescence, and drying of the leaf part directly adjacent to the sporophilic part. Similar trends associated with Chl loss were observed during sporangia maturation and leaf senescence in other fern species such as *Dryopteris affinis* or *Pteridium aquilinum* [14,36]. In the sporophilic part, an increase in Chl content (upper and lower side) was observed in stage II, which is a response to the increased demand for photosynthetic products during sporangia formation. Our studies confirm the presence of numerous, active chloroplasts in the perispore tissues (Figure 2 and Figure 7), which suggests their functional importance for spore formation in ferns. The greatest increase in Chl content in the sporophilic part occurred between SII and SIII in the upper part, while at the same time, a decrease in Chl was noted directly at the site of spore formation (bottom side). This may suggest a controlled degradation of Chl in the immediate vicinity of the sporangium and allocation of the degradation products to the upper part of the leaf, where increased Chl synthesis occurs. This is justified considering the changes occurring at this stage in the structure of the lower leaf surface (Figure 1 and Figure 2), associated with the development of dense tomentum, which hinders the absorption of light energy. Moreover, in seed plants, reproductive processes are often associated with changes in Chl metabolism and resource allocation, e.g., to generative organs [37]. However, it should be emphasized that in ferns, sporophyll or sporotrophophyll leaves often retain photosynthetic functions for a relatively long time. In *P. bifurcatum*, the complete loss of Chl associated with the aging of the sporophilic part and drying of the tissue took place only in the last stage of sporulation (SIV).

Figure 5 shows the dependence on the sporulation stage of flavonoid content in the tissue. The highest concentration of Flav was observed at the SII–SIII stage, on both the upper and lower surfaces of the sporotrophophyll, which indicates their participation in the process of spore formation and maturation. This is consistent with observations made on generative organs of seed plants, in which Flav content increases during meiosis [38]. The increase in Flav content is likely related to a protective function for forming spores, which are exposed to UV-B and high temperature–stressors crucial for plant reproduction. Flavonols are also present in spore protoplasm, capture reactive oxygen species, thus contributing to increased resistance to abiotic stresses [39]. The inability to determine Flav content at the SIV stage is due to the high degree of lignification of tissues, which prevents the detection of these compounds using nondestructive methods.

## 4. Materials and Methods

### 4.1. Plant Material

The study was conducted on 10-year-old sporophytes of *Platycerium bifurcatum* (Cav.) C. Chr..). The plants came from the collection of the University of National Education Commission, Krakow, and were grown for 6 months before the analyses in a greenhouse climate chamber under a natural photoperiod and controlled temperature conditions (22 °C/16 °C—day/night) as well as relative humidity of 60% (±5%). All plants were watered with tap water to a constant mass every 7 days, and once a month with standard Steiner medium. Measurements were performed in the trophophilic (TI–TIV) and sporophilic part of the leaf in the four stages of sporulation (SI–SIV) described earlier by Skoczowski et al. [20], in which the following were observed successively: juvenile sporangia during development (SI), maturing sporangia (SII, SIII), and mature sporangia releasing spores (SIV). Sporotrophophyll leaves without developed sporangia in the sporophilic part constituted the control–T0 and S0 (trophophilic and sporophilic part, respectively).

### 4.2. Anatomical Analysis

Cross sections were made manually in the sporophilic part of the leaf, approximately 2 cm from the apex. The anatomical analysis was performed in distilled water, representing cross-sections of the leaf lamina using a light microscope and a Nikon ECLIPSE Ni epifluorescence microscope (Nikon, Tokyo, Japan) equipped with Microscope Camera Digital Sight series DS-Fi1c and NIS Imaging, Nikon v. 4.11 software (Nikon, Tokyo, Japan). To induce autofluorescence, excitation in the UV and blue spectra was used.

### 4.3. Isothermal Calorimetry

Calorimetric measurements were performed on the leaves using an isothermal calorimetry (TAM III Thermometric 3101, TA Instruments, New Castle, DE, USA) at 20 °C, in the dark. The measurements were performed on 1 cm diameter discs cut from leaves at four development stages, both from the trophophilic and sporophilic parts. Due to the high degree of lignification of leaves at the SIV stage, calorimetric measurements were not performed there. The leaf discs were placed in measuring ampoules on paper discs moistened with distilled water. In the reference ampoule, water was contained in the same volume as that added to the experimental ampoules. Twenty-four-hour measurement of heat emission was carried out after 45 min of thermal stabilization. The results presented in the study are the mean of 8 independent biological replicates.

### 4.4. Analysis of Selected Plant Hormones

Selected phytohormones were analyzed following the method described by Płażek et al. [40]. Freeze-dried leaf fragments separated from sporophilic and trophophilic parts were extracted using 1 cm^3^ of a *P. bifurcatum* extraction buffer (methanol/water/formic acid in a 15:4:1 volume ratio) after the addition of an internal standard solution. The extraction was performed for 5 min at 30 Hz (MM400, Retch, Haan, Germany). The samples were then centrifuged at 22,000× *g* for 3 min (R32, Hettich, Tuttlingen, Germany), and the supernatant was collected. This extraction process was repeated two more times, and the resulting supernatants were combined, evaporated under nitrogen, and reconstituted in 5% methanol in 1 M formic acid. Cleanup was carried out using mixed-mode SPE cartridges (BondElutPlexa PCX, Agilent, Santa Clara, CA, USA), as outlined by Dziurka et al. [41]. All results were expressed in femtomoles (fmol) or micromoles per gram of dry weight (µmol g^−1^ DW).

Phytohormone analysis—including auxins, cytokinins, gibberellins, abscisic acid, and jasmonates—was performed using ultrahigh performance liquid chromatography (UHPLC) on an Agilent Infinity 1260 system coupled with a 6410 Triple Quad LC/MS equipped with an electrospray ionization (ESI) source (Agilent Technologies, Santa Clara, CA, USA). Separation was conducted on an Ascentis Express RP-Amide column (2.7 µm, 2.1 mm × 150 mm; Supelco, Bellefonte, PA, USA) with a linear gradient of water and acetonitrile containing 0.01% formic acid. The internal standards used were stable isotope-labeled versions of phytohormones, including: [^15^N_4_] dihydrozeatin, [^15^N_4_] kinetin, [^2^H_2_] gibberellin A4, A6, A5, [^2^H_5_] indole-3-acetic acid, and [^2^H_6_] cis,trans-abscisic acid (OlChemIm, Olomouc, Czech Republic).

Phytohormone profiling was performed using multiple reaction monitoring (MRM) and compared to pure and isotope-labeled standards of the target compounds. The identified hormone forms included: IAA, kinetin (KIN), zeatin (ZEA), active gibberellins (GA3, GA4, GA5, GA6, GA7), inactive gibberellins (GA8, GA9, GA20), active abscisic acid [(±)-cis,trans-ABA], inactive ABA glucosyl ester [(±)-cis,trans-ABA-glc], salicylic acid (SA), jasmonic acid (JA), and jasmonic acid methyl ester (JA-Met).

### 4.5. FT-Raman Spectroscopy Measurements

FT-Raman measurements were performed on both upper and bottom sides of sporophilic parts of leaves using a Nicolet NXR 9650 FT-Raman spectrometer (Thermo Fisher Scientific, Waltham, MA, USA) equipped with a Nd:YAG3+ laser emitting at 1064 nm and a germanium detector. All spectra were made at room temperature, at an aperture of 80 and a spectral resolution of 4 cm^−1^. They were collected in the range of 780–1800 cm^−1^, accumulated from 128 scans, and measured with the laser power of 0.4 W. Each presented spectrum was an average of 6–10 spectra. In order to extract the Raman signal from the registered results (containing the fluorescence background contribution originating from the intrinsic fluorescence of plant molecules), the baseline correction was performed. The spectra were normalized according to the most intense band, and the analysis was carried out using Omnic 8 and OriginPro 2017 software packages for Windows.

Two-dimensional Raman maps from the bottom parts of the tested leaves were obtained by moving the xy stage, where both x and y directions of the accessory were controlled by the software. The samples were irradiated with a focused laser beam of 100 mW and a diameter of about 0.1 mm. For all mapping measurements, the spectral resolution was constant and equal to 4 cm^−1^, and four scans were collected at each measured point. Raman mapping of leaves was performed at areas of 5.5 × 3.5 cm and with a step size of 100 µm. Bands related to -C=C- stretching vibration modes (1525 cm^−1^) were integrated and presented as 2-D maps colored according to Car content.

### 4.6. Chlorophyll and Flavonoid Content Measurement

The content of flavonoids and chlorophyll in the leaves was measured using a Dualex chlorophyllometer from FORCE-A, Orsay, France, at room temperature, according to the instructions for use issued by the manufacturer. Measurements were made on the leaves at four stages of development, on the upper and bottom sides of the leaf blade, in the sporophilic and trophophilic parts. The results presented in the study are the mean of 6 independent biological replicates.

### 4.7. Statistical Analysis

All results were analyzed using the Statistica 13 package (TIBCO Software, Palo Alto, CA, USA) using multivariate analysis of variance. Significance of differences was tested using Tukey’s test or Duncan’s test at a significance level of *p* ≤ 0.05.

## 5. Conclusions

This study revealed significant anatomical and metabolic differences between the sporulating and non-sporulating parts of a single leaf of the fern *P. bifurcatum*. Using the isothermal calorimetry method, for the first time in ferns, significant differences in metabolic activity within the leaf, accompanying the sporulation process and linked to the sporangium development stage, were proved. Furthermore, an increase in the number of photosynthetically active chloroplasts in perispore cells during the most intensive spore development phases was demonstrated.

The sporulation process is supported by an increase in the content of active gibberellins, particularly GA3, GA4, and GA6, and elevated concentrations of the auxin IBA, as well as ±ctABA and ABAGlc, in the sporophilic part of the sporotrophophyll leaf. However, in the trophophilic part, a slight increase in IBA and ABAGlc was observed, suggesting that ABAGlc was transported to the sporophilic part and hydrolyzed there, supporting the process of spore dehydration and protecting against osmotic stress. This indicates internal relocation of metabolites within a single leaf during sporulation.

Low cytokinin and elevated ABA levels indicate progressive tissue senescence in the trophophilic part of the leaf and ABA-dependent chlorophyll degradation in the final stage of sporulation. Raman analysis and 2D mapping revealed localized lignin accumulation and fluctuating carotenoid levels during spore maturation, linking these metabolic changes to progressive lignification and loss of tissue viability at the end of sporulation.

## Data Availability

Data are available from the authors upon request.

## Supplementary Materials

The following supporting information can be downloaded at: https://www.mdpi.com/article/10.3390/ijms26168084/s1. References [16,17,18,19,42] are cited in the Supplementary Materials.

## Abbreviations

±ctABA	(±)-cis, trans-abscisic acid
5CI-IAA	5-chloroindole-3-acetic acid
ABA-GE	abscisic acid glucose ester
BeA	benzoic acid
Car	carotenoids
Chl	chlorophyll
cZ	cis-zeatin
cZR	cis-zeatin riboside
DHZ	dihydrozeatin
DHZR	dihydrozeatin riboside
Flav	flavonols
GA3	gibberellin A3
GA4	gibberellin A4
GA5	gibberellin A5
GA6	gibberellin A6
GA9	gibberellin A9
IAA	indole-3-acetic acid
IBA	indole-3-butyric acid
iP	N6-isopentenyl adenine
iPR	N6-isopentenyl adenine riboside
JA	jasmonic acid
mT	meta-topolin
mTR	meta-topolin riboside
PSII	photosystem II
RC	reaction center of photosystem II
SA	salicylic acid
tZ	trans-zeatin
tZ7G	trans-Zeatin-7-glucoside
tZOG	glucosylated trans-zeatin
tZR	trans-zeatin riboside
