# Supplementary material for: Insight into the Sporulation Physiology of Elkhorn Fern: Metabolic, Hormonal, and Pigment Changes Within a Single Leaf of Platycerium bifurcatum
Source: Int J Mol Sci. 2025 Aug 21;26(16):8084. doi: 10.3390/ijms26168084 (PMC12386787; doi:10.3390/ijms26168084)
Supplement: Supplementary file 1 [file ijms-26-08084-s001.zip › ijms-3794063-supplementary.pdf]

Table S1. Endogenous phytohormone contents in *Platycerium bifurcatum* sporophylic and trophophylic parts of leaf. Δ (%T0) indicates percentage increase or decrease (negative numbers) compared to control stage (T0); nd - not detected; ns - no significant difference between the control stage (T0/S0) and developmental stages (TI-TIV/SI-SIV). Mean values from 3 independent biological replicates ±SD, marked with different letters in line, differ significantly according to Duncan's test, $p \leq 0.05$

**Phytohormone** (ng g^{-1} dry weight)	**Stage**									
	Sporophylic part					Trophophylic part				
	**T0**	**TI**	**TII**	**TIII**	**TIV**	**S0**	**SI**	**SII**	**SIII**	**SIV**
Gibberellin A3	27.51^{bc}	42.80^{b}	nd	23.94^{c}	77.30^{a}	nd	nd	nd	nd	22.15^{c}
	±11.40	±3.48	nd	±2.41	±18.46	nd	nd	nd	nd	±7.33
Gibberellin A4	11.98^{bc}	11.13^{bc}	13.23^{bc}	10.95^{bc}	22.39^{a}	14.21^{b}	10.83^{bc}	12.01^{bc}	8.71^{c}	13.87^{bc}
	±2.08	±2.89	±1.11	±1.94	±0.62	±4.31	±3.98	±3.20	±0.93	±3.61
Gibberellin A5	50.65^{bc}	69.50^{ab}	77.66^{a}	67.30^{ab}	50.60^{bc}	32.23^{cd}	32.90^{cd}	20.27^{d}	17.14^{d}	27.72^{cd}
	±6.92	±21.95	±10.15	±27.87	±9.97	±1.38	±5.21	±2.61	±4.53	±10.84
Gibberellin A6	4.23^{d}	18.25^{c}	20.95^{bc}	43.33^{a}	31.34^{b}	1.41^{e}	nd	nd	nd	nd
	±0.77	±6.55	±9.99	±0.84	±2.97	±0.20	nd	nd	nd	nd
Gibberellin A9	12.72^{cd}	12.29^{cd}	8.16^{d}	10.77^{cd}	10.89^{cd}	15.65^{bc}	12.45^{cd}	9.32^{cd}	21.66^{b}	30.93^{a}
	±2.93	±4.80	±1.04	±2.89	±2.37	±5.77	±1.14	±2.67	±1.97	±6.14
∑gibberellins	*107.10*	*153.98*	*120.00*	*156.29*	*192.52*	*63.50*	*56.18*	*41.60*	*47.50*	*94.66*
	±29.11	*±39.67*	*±22.29*	*±35.95*	*±34.39*	*±11.65*	*±10.33*	*±8.48*	*±7.44*	*±27.92*
Δgibberellins (%T0)	--------	**43.77**	**12.04**	**45.93**	**79.76**	--------	**ns**	**ns**	**-25.20**	**49.07**
IAA	26.52^{ab}	25.67^{ab}	18.44^{b}	26.09^{ab}	33.91^{a}	27.39^{ab}	23.13^{b}	25.27^{ab}	22.14^{b}	21.27^{b}
	±2.05	±9.53	±0.82	±4.78	±9.00	±3.66	±6.79	±2.89	±4.18	±2.10
5Cl-IAA	3.30^{e}	4.99^{de}	3.93^{de}	3.45^{e}	14.56^{a}	8.92^{bc}	13.20^{a}	6.86^{cd}	5.34^{de}	9.91^{b}
	±0.26	±1.32	±0.62	±0.99	±1.16	±2.73	±1.27	±1.21	±1.99	±2.64
IBA	22.68^{d}	30.31^{cd}	22.28^{d}	53.91^{b}	1111.16^{a}	21.73^{d}	32.37^{cd}	25.64^{cd}	21.55^{d}	45.06^{bc}
	±3.22	±4.90	±4.08	±5.04	±31.83	±4.56	±1.17	±4.56	±1.80	±9.26
∑auxins	*52.50*	*60.97*	*44.66*	*83.45*	*1159.63*	*58.05*	*68.70*	*57.77*	*49.04*	*76.24*
	±5.54	*±15.75*	*±5.52*	*±10.81*	*±41.98*	*±10.94*	*±9.23*	*±8.66*	*±7.97*	*±14.00*
Δauxins (%T0)	--------	**ns**	**ns**	**58.95**	**2108.82**	--------	**18.35**	**ns**	**-15.52**	**31.33**
*t*Z	3.66^{a}	3.87^{a}	3.35^{abc}	3.61^{a}	3.20^{abc}	3.34^{abc}	3.29^{abc}	2.77^{c}	3.00^{abc}	2.79^{bc}
	±0.30	±0.21	±0.45	±0.25	±0.20	±0.43	±0.44	±0.30	±0.25	±0.50
*t*ZR	6.72^{bc}	6.39^{bc}	5.79^{bc}	4.22^{bc}	4.10^{bc}	12.09^{b}	5.40^{bc}	3.86^{bc}	2.41^{c}	59.30^{a}
	±1.17	±1.41	±1.44	±0.30	±0.67	±5.38	±0.44	±1.08	±0.67	±13.74
*t*Z7G	2.67^{a}	2.95^{a}	2.38^{a}	3.03^{a}	3.04^{a}	2.82^{a}	2.76^{a}	2.61^{a}	2.54^{a}	2.63^{a}

	±0.41	±0.66	±0.10	±0.27	±0.27	±0.37	±0.42	±0.33	±0.12	±0.52
*t*ZOG	17.24^{a}	15.60^{a}	6.11^{bc}	3.51^{d}	11.45^{b}	12.08^{b}	10.74^{bc}	9.24^{bc}	2.96^{d}	5.58^{c}
	±1.61	±3.41	±0.66	±0.19	±1.50	±1.46	±2.05	±1.21	±0.13	±0.48
∑*t*Z-types	*30.29*	*28.81*	*17.64*	*14.37*	*21.79*	*30.34*	*22.20*	*18.49*	*10.91*	*70.30*
	±3.49	*±5.70*	*±2.65*	*±1.01*	*±2.64*	*±7.64*	*±3.36*	*±2.92*	*±1.17*	*±15.25*
Δ*t*Z-types (%T0)	--------	**ns**	**-41.76**	**-52.56**	**-28.06**	--------	**ns**	**ns**	**-64.04**	**131.71**
*c*Z	1.91^{a}	2.15^{a}	1.91^{a}	2.39^{a}	2.29^{a}	2.09^{a}	1.98^{a}	1.84^{a}	1.85^{a}	2.13^{a}
	±0.30	±0.63	±0.14	±0.26	±0.08	±0.39	±0.44	±0.17	±0.15	±0.41
*c*ZR	3.43^{a}	3.82^{a}	2.93^{a}	3.10^{a}	2.82^{a}	3.35a	3.39^{a}	3.44^{a}	3.19^{a}	3.07^{a}
	±0.39	±1.97	±0.42	±0.58	±1.13	±0.54	±0.69	±0.72	±0.42	±0.44
∑*c*Z-types	*5.34*	*5.97*	*4.84*	*5.50*	*5.11*	*5.44*	*5.36*	*5.29*	*5.04*	*5.20*
	±0.69	*±2.60*	*±0.56*	*±0.85*	*±1.21*	*±0.93*	*±1.13*	*±0.89*	*±0.57*	*±0.85*
Δ*c*Z-types	--------	**ns**	**ns**	**ns**	**ns**	--------	**ns**	**ns**	**ns**	**ns**
DHZ	1.53^{a}	1.23^{a}	1.14^{a}	1.63^{a}	1.87^{a}	1.23^{a}	1.27^{a}	1.07^{a}	1.13^{a}	1.33^{a}
	±0.28	±0.23	±0.18	±0.37	±0.08	±0.22	±0.14	±0.17	±0.05	±0.38
DHZR	3.45^{c}	4.60^{c}	3.30^{c}	3.69^{c}	13.56^{b}	3.94^{c}	2.79^{c}	3.19^{c}	2.55^{c}	18.63^{a}
	±0.41	±1.34	±0.56	±0.76	±1.97	±0.82	±0.40	±0.33	±0.47	±4.07
∑DHZ-types	*1.66*	*2.92*	*2.22*	*2.66*	*7.72*	*1.72*	*2.03*	*2.13*	*1.84*	*9.98*
	±0.69	*±1.57*	*±0.74*	*±1.13*	*±2.05*	*±1.04*	*±0.54*	*±0.50*	*±0.53*	*±4.45*
ΔDHZ-types (%T0)	--------	**ns**	**ns**	**ns**	**210.00**	--------	**ns**	**ns**	**ns**	**285.33**
iP	19.28^{ab}	20.59^{ab}	14.43^{ac}	20.14^{ab}	21.31^{a}	19.10^{ab}	17.78^{abc}	10.58^{c}	15.90^{c}	16.48^{c}
	±2.78	±4.06	±0.18	±1.63	±2.47	±2.39	±2.55	±0.50	±1.14	±3.44
iPR	24.12^{bc}	26.12^{bc}	17.83^{de}	27.26^{bc}	16.22^{e}	34.13^{a}	23.96^{bc}	22.42^{cd}	28.40^{b}	24.90^{bc}
	±0.86	±3.30	±3.80	±1.20	±0.91	±4.18	±0.29	±1.04	±0.79	±6.44
∑iP-types	*43.40*	*46.71*	*32.25*	*47.40*	*37.53*	*53.24*	*41.74*	*33.00*	*44.30*	*41.38*
	±3.64	*±7.37*	*±3.97*	*±2.82*	*±3.38*	*±6.58*	*±2.84*	*±1.55*	*±1.93*	*±9.88*
ΔiP-types (%T0)	--------	**ns**	**-25.34**	**ns**	**-13.52**	--------	**-21.60**	**-38.02**	**-16.79**	**-22.28**
*m*T	5.03^{a}	5.43^{a}	4.46^{a}	5.62^{a}	5.47^{a}	5.08^{a}	5.06^{a}	4.87^{a}	4.66^{a}	4.93^{a}
	±0.91	±1.34	±0.10	±0.81	±0.55	±0.51	±0.70	±0.30	±0.56	±1.21
mTR	nd	nd	nd	nd	nd	nd	2.37	nd	nd	nd
	nd	nd	nd	nd	nd	nd	0.44	nd	nd	nd
∑*m*T-types	*5.03*	*5.43*	*4.46*	*5.62*	*5.47*	*5.08*	*7.43*	*4.87*	*4.66*	*4.93*
	±0.91	*±1.34*	*±0.10*	*±0.81*	*±0.55*	*±0.51*	*1.14*	*±0.30*	*±0.56*	*±1.21*
Δ*m*T-types (%T0)	--------	**ns**	**ns**	**ns**	**ns**	--------	**ns**	**ns**	**ns**	**ns**

∑CKs total	*85.72*	*89.83*	*61.40*	*75.56*	*77.62*	*95.82*	*78.76*	*63.78*	*66.74*	*131.79*
	±9.42	*±18.58*	*±8.03*	*±6.62*	*±9.84*	*±16.70*	*±9.01*	*±6.16*	*±4.76*	*±31.64*
ΔCKs total (%T0)	--------	**ns**	**-29.06**	**-12.70**	**-10.32**	--------	**-18.54**	**-34.04**	**-30.97**	**36.30**
±ctABA	113.03^{b}	164.93^{b}	126.45^{b}	159.46^{b}	600.33^{a}	161.82^{b}	217.32^{b}	177.44^{b}	95.85^{b}	95.30^{b}
	±9.36	±20.16	±197.03	±25.79	±12.18	±31.32	±12.67	±30.67	±9.36	±20.16
ABAGlc	61.01^{e}	66.44^{e}	40.37^{e}	74.26^{de}	143.66^{b}	83.55^{d}	125.74^{bc}	88.17^{cd}	126.01^{bc}	254.90^{a}
	±5.11	±10.09	±47.97	±8.62	±6.95	±7.82	±17.10	±36.97	±5.11	±10.09
∑ABA	174.03	231.37	166.82	233.72	743.99	245.36	343.06	265.61	221.87	350.20
	±14.47	±30.25	±245.00	±34.41	±19.13	±39.14	±29.77	±67.64	±14.47	±30.25
ΔABA (%T0)	--------	**ns**	**ns**	**ns**	**327.51**	--------	**39.82**	**ns**	**-9.57**	**42.73**
JA	153.43^{cd}	175.20^{c}	65.52^{e}	40.66^{e}	90.20^{de}	262.68^{b}	391.69^{a}	165.58^{c}	93.28^{de}	35.73^{e}
	±8.54	±9.75	±13.25	±64.07	±90.15	±33.04	±7.96	±5.61	±8.54	±9.75
ΔJA (%T0)	--------	**ns**	**-57.30**	**-73.50**	**-41.21**	--------	**49.11**	**-36.96**	**-64.49**	**-86.40**
SA	2595.35^{a}	497.64^{de}	602.79^{d}	337.90^{e}	590.68^{d}	1526.19^{b}	550.65^{de}	947.20^{c}	362.21^{e}	493.40^{de}
	±72.36	±34.34	±42.75	±187.55	±128.47	±33.45	±74.72	±108.68	±72.36	±34.34
ΔSA (%T0)	--------	**-80.82**	**-76.77**	**-86.98**	**-77.24**	--------	**-63.92**	**-37.94**	**-76.27**	**-67.67**
BeA	5254.00^{bc}	7416.07^{b}	3937.88^{c}	3984.16^{c}	5282.21^{bc}	4530.62^{c}	5650.02^{bc}	14589.29^{a}	3914.13^{c}	3329.63^{c}
	±431.12	±648.49	±631.70	±1176.65	±632.16	±1604.63	±625.52	±225.37	±431.12	±648.49
ΔBeA (%T0)	--------	**41.15**	**ns**	**ns**	**ns**	--------	**ns**	**222.01**	**ns**	**ns**

Table S2. The characterization of chemical compounds assigned to the particular bands in the FT-Raman spectra obtained from the bottom and upper sides of sporophilic parts of *Platycerium bifurcatum* leaves.

Peak numer	Wave number (cm^{-1})	Components	References
1	1005	carotenoid (tetraterpenes)	Baranska et al. 2005
2	1093	polysaccharides (cellulose)	Schulz and Baranska 2005
3	1121	polysaccharides, disaccharides (celulose, sucrose)	Schulz and Baranska 2007 Barron et al. 2006
4	1159	carotenoids (tetraterpenes)	Baranska et al. 2005, Schrader et al. 1999
5	1293	lipids, fatty acids;	Schulz and Baranska 2007
6	1374	lipids, fatty acids;	Schulz and Baranska 2007
7	1440	lipids, fatty acids	Schulz and Baranska 2007
8	1525	carotenoids (tetraterpenes)	Schulz and Baranska 2007
9	1607	chlorophylls, flavonoids	Schrader et al. 1999, Schulz and Baranska 2007
10	1631	lipids, fatty acids	Schulz and Baranska 2007

1. Schulz, H.; Baranska, M.; Baranski, R. Potential of NIR-FT-Raman spectroscopy in natural carotenoid analysis. Biopolymers 2005, 77, 212–221. https://doi.org/10.1002/bip.20215.
2. Baranska, M.; Schulz, H.; Baranski, R.; Nothnagel, T.; Christensen, L.P. In situ simultaneous analysis of polyacetylenes, carotenoids and polysaccharides in carrot roots. J. Agric. Food Chem. 2005, 53, 6565–6571. https://doi.org/10.1021/jf0510440.
3. Schrader, B.; Klump, H.H.; Schenzel, K.; Schulz, H. Non-destructive NIR FT Raman analysis of plants. J. Mol. Struct. 1999, 509, 201–212. https://doi.org/10.1016/S0022-2860(99)00221-5.
4. Schulz, H.; Baranska, M. Identification and quantification of valuable plant substances by IR and Raman spectroscopy. Vib. Spectrosc. 2007, 43, 13–25.
5. Barron, C.; Robert, P.; Guillon, F.; Saulnier, L.; Rouau, X. Structural heterogeneity of wheat arabinoxylans revealed by Raman spectroscopy. Carbohydr. Res. 2006, 341, 1186–1191.
